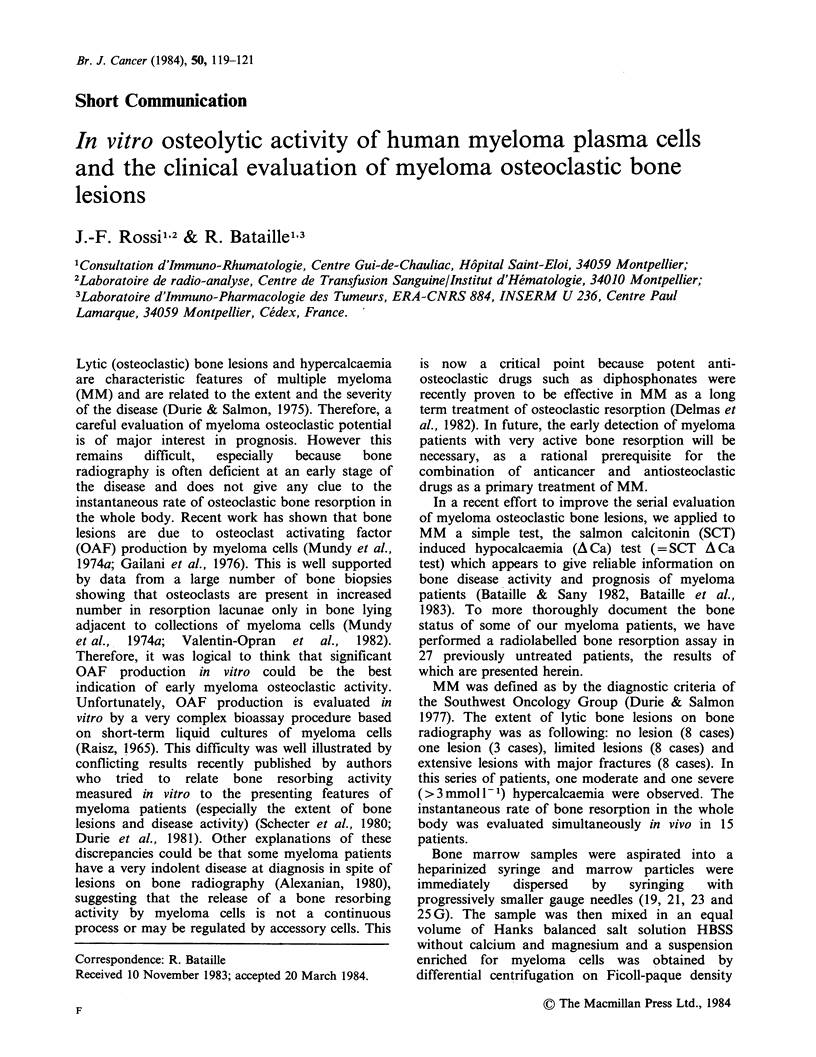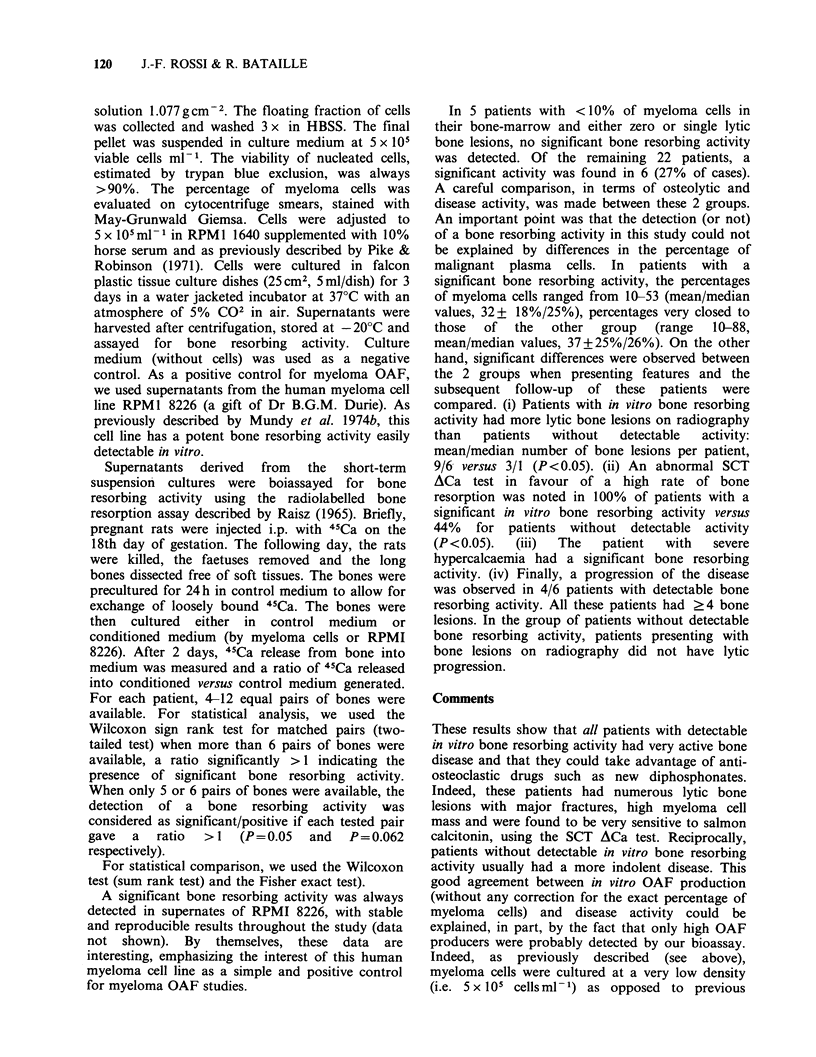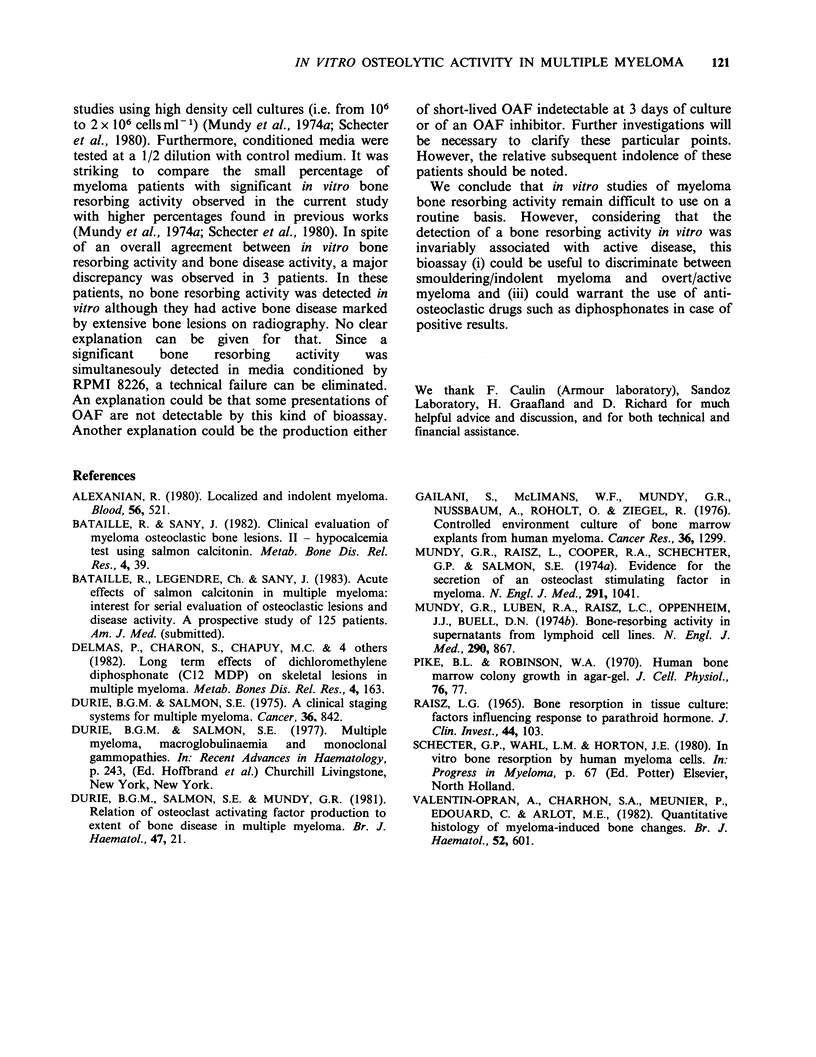# In vitro osteolytic activity of human myeloma plasma cells and the clinical evaluation of myeloma osteoclastic bone lesions.

**DOI:** 10.1038/bjc.1984.147

**Published:** 1984-07

**Authors:** J. F. Rossi, R. Bataille


*Br. J. Cancer* (1984), **50**, 119–121

### Short Communication

### *In vitro* osteolytic activity of human myeloma plasma cells and the clinical evaluation of myeloma osteoclastic bone lesions

J.-F. Rossi[1,2] & R. Bataille[1,3]

[1]*Consultation d'Immuno-Rhumatologie, Centre Gui-de-Chauliac, Hôpital Saint-Eloi, 34059 Montpellier;* [2]*Laboratoire de radio-analyse, Centre de Transfusion Sanguine/Institut d'Hématologie, 34010 Montpellier;* [3]*Laboratoire d'Immuno-Pharmacologie des Tumeurs, ERA-CNRS 884, INSERM U 236, Centre Paul Lamarque, 34059 Montpellier, Cédex, France.*

Lytic (osteoclastic) bone lesions and hypercalcaemia are characteristic features of multiple myeloma (MM) and are related to the extent and the severity of the disease (Durie & Salmon, 1975). Therefore, a careful evaluation of myeloma osteoclastic potential is of major interest in prognosis. However this remains difficult, especially because bone radiography is often deficient at an early stage of the disease and does not give any clue to the instantaneous rate of osteoclastic bone resorption in the whole body. Recent work has shown that bone lesions are due to osteoclast activating factor (OAF) production by myeloma cells (Mundy *et al.*, 1974*a*; Gailani *et al.*, 1976). This is well supported by data from a large number of bone biopsies showing that osteoclasts are present in increased number in resorption lacunae only in bone lying adjacent to collections of myeloma cells (Mundy *et al.*, 1974*a*; Valentin-Opran *et al.*, 1982). Therefore, it was logical to think that significant OAF production *in vitro* could be the best indication of early myeloma osteoclastic activity. Unfortunately, OAF production is evaluated *in vitro* by a very complex bioassay procedure based on short-term liquid cultures of myeloma cells (Raisz, 1965). This difficulty was well illustrated by conflicting results recently published by authors who tried to relate bone resorbing activity measured *in vitro* to the presenting features of myeloma patients (especially the extent of bone lesions and disease activity) (Schecter *et al.*, 1980; Durie *et al.*, 1981). Other explanations of these discrepancies could be that some myeloma patients have a very indolent disease at diagnosis in spite of lesions on bone radiography (Alexanian, 1980), suggesting that the release of a bone resorbing activity by myeloma cells is not a continuous process or may be regulated by accessory cells. This is now a critical point because potent anti-osteoclastic drugs such as diphosphonates were recently proven to be effective in MM as a long term treatment of osteoclastic resorption (Delmas *et al.*, 1982). In future, the early detection of myeloma patients with very active bone resorption will be necessary, as a rational prerequisite for the combination of anticancer and antiosteoclastic drugs as a primary treatment of MM.

In a recent effort to improve the serial evaluation of myeloma osteoclastic bone lesions, we applied to MM a simple test, the salmon calcitonin (SCT) induced hypocalcaemia ($\Delta$Ca) test (=SCT $\Delta$Ca test) which appears to give reliable information on bone disease activity and prognosis of myeloma patients (Bataille & Sany 1982, Bataille *et al.*, 1983). To more thoroughly document the bone status of some of our myeloma patients, we have performed a radiolabelled bone resorption assay in 27 previously untreated patients, the results of which are presented herein.

MM was defined as by the diagnostic criteria of the Southwest Oncology Group (Durie & Salmon 1977). The extent of lytic bone lesions on bone radiography was as following: no lesion (8 cases) one lesion (3 cases), limited lesions (8 cases) and extensive lesions with major fractures (8 cases). In this series of patients, one moderate and one severe ($>3\,\mathrm{mmol\,l^{-1}}$) hypercalcaemia were observed. The instantaneous rate of bone resorption in the whole body was evaluated simultaneously *in vivo* in 15 patients.

Bone marrow samples were aspirated into a heparinized syringe and marrow particles were immediately dispersed by syringing with progressively smaller gauge needles (19, 21, 23 and 25 G). The sample was then mixed in an equal volume of Hanks balanced salt solution HBSS without calcium and magnesium and a suspension enriched for myeloma cells was obtained by differential centrifugation on Ficoll-paque density

Correspondence: R. Bataille
Received 10 November 1983; accepted 20 March 1984.

© The Macmillan Press Ltd., 1984

solution 1.077 g $cm^{-2}$. The floating fraction of cells was collected and washed 3 × in HBSS. The final pellet was suspended in culture medium at $5 \times 10^5$ viable cells $ml^{-1}$. The viability of nucleated cells, estimated by trypan blue exclusion, was always >90%. The percentage of myeloma cells was evaluated on cytocentrifuge smears, stained with May-Grunwald Giemsa. Cells were adjusted to $5 \times 10^5 ml^{-1}$ in RPMI 1640 supplemented with 10% horse serum and as previously described by Pike & Robinson (1971). Cells were cultured in falcon plastic tissue culture dishes (25 $cm^2$, 5 ml/dish) for 3 days in a water jacketed incubator at 37°C with an atmosphere of 5% $CO^2$ in air. Supernatants were harvested after centrifugation, stored at −20°C and assayed for bone resorbing activity. Culture medium (without cells) was used as a negative control. As a positive control for myeloma OAF, we used supernatants from the human myeloma cell line RPMI 8226 (a gift of Dr B.G.M. Durie). As previously described by Mundy *et al.* 1974*b*, this cell line has a potent bone resorbing activity easily detectable *in vitro*.

Supernatants derived from the short-term suspension cultures were boiassayed for bone resorbing activity using the radiolabelled bone resorption assay described by Raisz (1965). Briefly, pregnant rats were injected i.p. with $^{45}Ca$ on the 18th day of gestation. The following day, the rats were killed, the faetuses removed and the long bones dissected free of soft tissues. The bones were precultured for 24 h in control medium to allow for exchange of loosely bound $^{45}Ca$. The bones were then cultured either in control medium or conditioned medium (by myeloma cells or RPMI 8226). After 2 days, $^{45}Ca$ release from bone into medium was measured and a ratio of $^{45}Ca$ released into conditioned *versus* control medium generated. For each patient, 4–12 equal pairs of bones were available. For statistical analysis, we used the Wilcoxon sign rank test for matched pairs (two-tailed test) when more than 6 pairs of bones were available, a ratio significantly >1 indicating the presence of significant bone resorbing activity. When only 5 or 6 pairs of bones were available, the detection of a bone resorbing activity was considered as significant/positive if each tested pair gave a ratio >1 ($P = 0.05$ and $P = 0.062$ respectively).

For statistical comparison, we used the Wilcoxon test (sum rank test) and the Fisher exact test).

A significant bone resorbing activity was always detected in supernates of RPMI 8226, with stable and reproducible results throughout the study (data not shown). By themselves, these data are interesting, emphasizing the interest of this human myeloma cell line as a simple and positive control for myeloma OAF studies.

In 5 patients with <10% of myeloma cells in their bone-marrow and either zero or single lytic bone lesions, no significant bone resorbing activity was detected. Of the remaining 22 patients, a significant activity was found in 6 (27% of cases). A careful comparison, in terms of osteolytic and disease activity, was made between these 2 groups. An important point was that the detection (or not) of a bone resorbing activity in this study could not be explained by differences in the percentage of malignant plasma cells. In patients with a significant bone resorbing activity, the percentages of myeloma cells ranged from 10–53 (mean/median values, 32 ± 18%/25%), percentages very closed to those of the other group (range 10–88, mean/median values, 37 ± 25%/26%). On the other hand, significant differences were observed between the 2 groups when presenting features and the subsequent follow-up of these patients were compared. (i) Patients with *in vitro* bone resorbing activity had more lytic bone lesions on radiography than patients without detectable activity: mean/median number of bone lesions per patient, 9/6 *versus* 3/1 ($P < 0.05$). (ii) An abnormal SCT ΔCa test in favour of a high rate of bone resorption was noted in 100% of patients with a significant *in vitro* bone resorbing activity *versus* 44% for patients without detectable activity ($P < 0.05$). (iii) The patient with severe hypercalcaemia had a significant bone resorbing activity. (iv) Finally, a progression of the disease was observed in 4/6 patients with detectable bone resorbing activity. All these patients had ≥4 bone lesions. In the group of patients without detectable bone resorbing activity, patients presenting with bone lesions on radiography did not have lytic progression.

### Comments

These results show that *all* patients with detectable *in vitro* bone resorbing activity had very active bone disease and that they could take advantage of anti-osteoclastic drugs such as new diphosphonates. Indeed, these patients had numerous lytic bone lesions with major fractures, high myeloma cell mass and were found to be very sensitive to salmon calcitonin, using the SCT ΔCa test. Reciprocally, patients without detectable *in vitro* bone resorbing activity usually had a more indolent disease. This good agreement between *in vitro* OAF production (without any correction for the exact percentage of myeloma cells) and disease activity could be explained, in part, by the fact that only high OAF producers were probably detected by our bioassay. Indeed, as previously described (see above), myeloma cells were cultured at a very low density (i.e. $5 \times 10^5$ cells $ml^{-1}$) as opposed to previous studies using high density cell cultures (i.e. from $10^6$ to $2 \times 10^6$ cells $ml^{-1}$) (Mundy *et al.*, 1974*a*; Schecter *et al.*, 1980). Furthermore, conditioned media were tested at a 1/2 dilution with control medium. It was striking to compare the small percentage of myeloma patients with significant *in vitro* bone resorbing activity observed in the current study with higher percentages found in previous works (Mundy *et al.*, 1974*a*; Schecter *et al.*, 1980). In spite of an overall agreement between *in vitro* bone resorbing activity and bone disease activity, a major discrepancy was observed in 3 patients. In these patients, no bone resorbing activity was detected *in vitro* although they had active bone disease marked by extensive bone lesions on radiography. No clear explanation can be given for that. Since a significant bone resorbing activity was simultanesouly detected in media conditioned by RPMI 8226, a technical failure can be eliminated. An explanation could be that some presentations of OAF are not detectable by this kind of bioassay. Another explanation could be the production either of short-lived OAF indetectable at 3 days of culture or of an OAF inhibitor. Further investigations will be necessary to clarify these particular points. However, the relative subsequent indolence of these patients should be noted.

We conclude that *in vitro* studies of myeloma bone resorbing activity remain difficult to use on a routine basis. However, considering that the detection of a bone resorbing activity *in vitro* was invariably associated with active disease, this bioassay (i) could be useful to discriminate between smouldering/indolent myeloma and overt/active myeloma and (iii) could warrant the use of anti-osteoclastic drugs such as diphosphonates in case of positive results.

We thank F. Caulin (Armour laboratory), Sandoz Laboratory, H. Graafland and D. Richard for much helpful advice and discussion, and for both technical and financial assistance.

### References

ALEXANIAN, R. (1980). Localized and indolent myeloma. *Blood*, **56**, 521.

BATAILLE, R. & SANY, J. (1982). Clinical evaluation of myeloma osteoclastic bone lesions. II – hypocalcemia test using salmon calcitonin. *Metab. Bone Dis. Rel. Res.*, **4**, 39.

BATAILLE, R., LEGENDRE, Ch. & SANY, J. (1983). Acute effects of salmon calcitonin in multiple myeloma: interest for serial evaluation of osteoclastic lesions and disease activity. A prospective study of 125 patients. *Am. J. Med.* (submitted).

DELMAS, P., CHARON, S., CHAPUY, M.C. & 4 others (1982). Long term effects of dichloromethylene diphosphonate (C12 MDP) on skeletal lesions in multiple myeloma. *Metab. Bones Dis. Rel. Res.*, **4**, 163.

DURIE, B.G.M. & SALMON, S.E. (1975). A clinical staging systems for multiple myeloma. *Cancer*, **36**, 842.

DURIE, B.G.M. & SALMON, S.E. (1977). Multiple myeloma, macroglobulinaemia and monoclonal gammopathies. *In: Recent Advances in Haematology*, p. 243, (Ed. Hoffbrand *et al.*) Churchill Livingstone, New York, New York.

DURIE, B.G.M., SALMON, S.E. & MUNDY, G.R. (1981). Relation of osteoclast activating factor production to extent of bone disease in multiple myeloma. *Br. J. Haematol.*, **47**, 21.

GAILANI, S., McLIMANS, W.F., MUNDY, G.R., NUSSBAUM, A., ROHOLT, O. & ZIEGEL, R. (1976). Controlled environment culture of bone marrow explants from human myeloma. *Cancer Res.*, **36**, 1299.

MUNDY, G.R., RAISZ, L., COOPER, R.A., SCHECHTER, G.P. & SALMON, S.E. (1974*a*). Evidence for the secretion of an osteoclast stimulating factor in myeloma. *N. Engl. J. Med.*, **291**, 1041.

MUNDY, G.R., LUBEN, R.A., RAISZ, L.C., OPPENHEIM, J.J., BUELL, D.N. (1974*b*). Bone-resorbing activity in supernatants from lymphoid cell lines. *N. Engl. J. Med.*, **290**, 867.

PIKE, B.L. & ROBINSON, W.A. (1970). Human bone marrow colony growth in agar-gel. *J. Cell. Physiol.*, **76**, 77.

RAISZ, L.G. (1965). Bone resorption in tissue culture: factors influencing response to parathroid hormone. *J. Clin. Invest.*, **44**, 103.

SCHECTER, G.P., WAHL, L.M. & HORTON, J.E. (1980). In vitro bone resorption by human myeloma cells. *In: Progress in Myeloma*, p. 67 (Ed. Potter) Elsevier, North Holland.

VALENTIN-OPRAN, A., CHARHON, S.A., MEUNIER, P., EDOUARD, C. & ARLOT, M.E., (1982). Quantitative histology of myeloma-induced bone changes. *Br. J. Haematol.*, **52**, 601.